# Joint QTL Linkage Mapping for Multiple-Cross Mating Design Sharing One Common Parent

**DOI:** 10.1371/journal.pone.0017573

**Published:** 2011-03-15

**Authors:** Huihui Li, Peter Bradbury, Elhan Ersoz, Edward S. Buckler, Jiankang Wang

**Affiliations:** 1 Institute of Crop Science, The National Key Facility for Crop Gene Resources and Genetic Improvement, and CIMMYT China, Chinese Academy of Agricultural Sciences, Beijing, China; 2 Institute for Genomic Diversity, Cornell University, Ithaca, New York, United States of America; 3 United States Department of Agriculture-Agricultural Research Service, Robert W. Holley Center for Agriculture and Health, Ithaca, New York, United States of America; University of California Davis, United States of America

## Abstract

**Background:**

Nested association mapping (NAM) is a novel genetic mating design that combines the advantages of linkage analysis and association mapping. This design provides opportunities to study the inheritance of complex traits, but also requires more advanced statistical methods. In this paper, we present the detailed algorithm of a QTL linkage mapping method suitable for genetic populations derived from NAM designs. This method is called joint inclusive composite interval mapping (JICIM). Simulations were designed on the detected QTL in a maize NAM population and an *Arabidopsis* NAM population so as to evaluate the efficiency of the NAM design and the JICIM method.

**Principal Findings:**

Fifty-two QTL were identified in the maize population, explaining 89% of the phenotypic variance of days to silking, and nine QTL were identified in the *Arabidopsis* population, explaining 83% of the phenotypic variance of flowering time. Simulations indicated that the detection power of these identified QTL was consistently high, especially for large-effect QTL. For rare QTL having significant effects in only one family, the power of correct detection within the 5 cM support interval was around 80% for 1-day effect QTL in the maize population, and for 3-day effect QTL in the *Arabidopsis* population. For smaller-effect QTL, the power diminished, e.g., it was around 50% for maize QTL with an effect of 0.5 day. When QTL were linked at a distance of 5 cM, the likelihood of mapping them as two distinct QTL was about 70% in the maize population. When the linkage distance was 1 cM, they were more likely mapped as one single QTL at an intermediary position.

**Conclusions:**

Because it takes advantage of the large genetic variation among parental lines and the large population size, NAM is a powerful multiple-cross design for complex trait dissection. JICIM is an efficient and specialty method for the joint QTL linkage mapping of genetic populations derived from the NAM design.

## Introduction

QTL mapping based on biparental populations has become a routine approach for genetic studies of complex traits in plants and animals. In most biparental populations, recombination has not had enough time to shuffle the genome into small fragments, and QTL are generally located in large chromosomal regions [1], [2]. In addition, linkage mapping based on biparental populations can only identify QTL from the phenotypic diversity generated from the controlled cross. In contrast, association mapping is based on natural populations, searching for genotype to phenotype correlations in unrelated individuals. Though more rapid and cost-effective compared with linkage mapping, association mapping is heavily dependent on population structure, which is normally unknown [3]. Nested association mapping (NAM) population derived from a multiple-cross mating design sharing one common parent was therefore proposed to enable high power and high resolution through joint linkage and association analysis, and to provide a broader genetic resource for quantitative trait analysis [4]. As an example, one NAM population in maize was recently reported [5], [6].

Several methods have been proposed for QTL mapping on populations derived from multiple strain crosses. Rebaï and Goffinet adapted the linear regression method to the case of a diallel cross between four inbred lines [7], and afterwards presented a general linear model (GLM) for QTL mapping by combining different populations derived from diallel designs [8]. Xu [9] proposed a fixed model and a random model for multiple independent families based on the weighted least square method of linear model. By using mixture model, Liu and Zeng [10] extended composite interval mapping (CIM; [11]) to the algorithm of multiple inbred lines, and Jourjon et al. [12] implemented the CIM and iterative QTL mapping in MCQTL software to perform QTL mapping either for independent families or for families from the diallel design. Jannink and Jansen [13] developed a multiple-QTL model for multiple related populations derived from the diallel of pairwise crosses among three inbred parents. Based on the mixed model, Crepieux et al. [14] developed a two-step IBD (identical by descent) variance component approach for breeding populations obtained from inbred parents. On the Bayesian perspective, Yi and Xu [15], [16] developed a Bayesian method implemented via the Markov chain Monte Carlo algorithm for mapping QTL using complicated multiple line crosses. Fang et al. [17] extended Bayesian model selection and the Bayesian shrinkage estimation approach to multiple independent crosses.

For the NAM design, Yu et al. [4] used the principle of association mapping to investigate its genetic and statistical properties by simulation experiments. As recombination information is not used, association mapping cannot give a position estimation of the identified QTL, which is essential for gene fine-mapping and map-based cloning, especially under moderate marker density. More recently, Hayashi and Iwata [18] simulated a total of 2000 individuals in a NAM population and 84 evenly distributed markers in the whole genome to evaluate a Bayesian method for QTL mapping. Nevertheless, as Xu and Jia [19] pointed out, Bayesian models still have problems detecting QTL in real populations.

Spearheaded by the maize NAM population of Buckler et al. [5], such augmented study designs are gaining more acknowledgement and application in breeding and genetics studies for various species. Despite the fact that more data and results in this framework are quickly becoming available, there is no algorithm specifically designed to analyze data from such studies. This is to emphasize the fact that although, several methods have been proposed for simultaneous analysis of such networked multi-population QTL data [7]–[10], [12]–[17], none of these methods are optimized to take full advantage of the reference design cases. In this paper, we present the algorithm of joint inclusive composite interval mapping (JICIM), which is specialty for NAM design and has been adopted in the paper published in Science by Buckler et al. [5]. We then used the QTL identified by JICIM in the real maize and *Arabidopsis* NAM populations to define genetic models in simulation experiments, so as to evaluate the statistical power and reliability of the identified QTL and the efficiency of JICIM to detect rare QTL in the NAM design. By rare QTL, in this study we mean a QTL that segregates in just one family, i.e., there are two alternative alleles having identifiable genetic effects on a trait of interest in the segregating family. In other families, either the same allele is located at the rare QTL chromosomal position, or the two alleles show no significant difference in their genetic effects on the trait of interest. In contrast, QTL that segregate in a number of families are called common QTL.

## Results

### Mapping results in two NAM populations

For days to silking in the maize population, 52 QTL were identified by JICIM in their one-LOD support intervals across the whole genome (Table S1), with a LOD threshold of 12.26 to guard against more than two false positives, i.e., GWER(2) = 12.26 [20]. Among the 52 QTL, the top 30 QTL (denoted as *qZ*1-*qZ*30, where *q* stands for QTL and *Z* for *Zea mays* L.) explain 84% of the total phenotypic variance, and were used to demonstrate the genetic architecture of maize flowering time by Buckler et al. [5]. The chromosomal distribution and effect size of the identified QTL across the 25 families (Fig. S1) supported the conclusion that large differences in flowering time among inbred maize lines are caused by the cumulative effects of numerous QTL [5]. Among all the detected QTL, there is no rare QTL that has significant additive effect in only one family.

**Figure 1:**
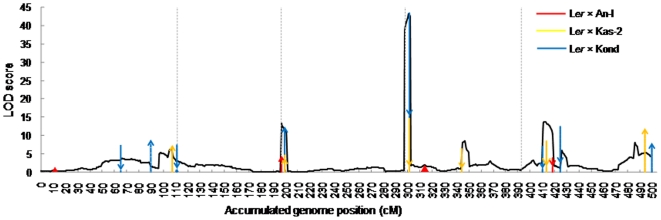
The one-dimensional scanning LOD profile of JICIM in the *Arabidopsis* population. The scanning step was 1 cM. Arrow size and direction represent the approximate effect size and direction of the identified QTL based on individual family mapping. Different colors of arrows indicate QTL identified in different families by individual family mapping.

For flowering time in the *Arabidopsis* population [21] and when the LOD threshold of GWER(2) = 3.30, nine QTL (denoted as *qA*1-*qA*9, where *A* stands for *Arabidopsis*) are identified in one-LOD support interval across the whole genome, explaining 83% of the phenotypic variance (Table 1 and Fig. 1). Flowering time is a well studied trait in *Arabidopsis*, and numerous flowering time genes have been identified (cloned) in *Arabidopsis*, based on mutation analysis [22]–[24], but that the relationship between these genes and the QTL underlying variation in mapping populations derived from accession crosses is unclear. None-the-less, there are previous reports in the literature identifying the same 9 QTL that were detected by JICIM, albeit there seems to be clusters of candidate genes underlying these QTL. For instance, when the positions of the QTL detected were compared to the results from the candidate gene association mapping study of Ehrenreich et al. [25], there seems to be a cluster of three important flowering time candidate genes, i.e., FRI, LD and GA1 corresponding to the position underlying the largest QTL around marker *FRI* from the FRIGIDA gene in this study. This locus is significant in all the three families, and explains 53.84% of the phenotypic variance. A table of candidate genes identified to be significant in the association study by Ehrenreich et al. [25] and Brachi et al. [26] that correspond to the QTL intervals detected in this study is included for the purposes of linking this study to the rest of the *Arabidopsis* literature (Table 1).

**Table 1:** Nine flowering time QTL identified in the *Arabidopsis* NAM population by JICIM.

QTL	Chr.	Pos. (cM)	LOD score	PVE (%)^a^	Left marker (cM)^b^	Right marker (cM)^b^	Additive genetic effect (day) in each family c			PVE (%) in each family			Flowering genes around QTL^d^	
							L*er*×An-l	L*er*×Kas-2	L*er*×Kond	L*er*×An-l	L*er*×Kas-2	L*er*×Kond	Brachi et al. [26]	Ehrenreich et al. [25]
*qA*1	1	66.0	3.75	3.92	CIW1* (65.2)	F6D8.94 (69.4)	−0.28	0.60	**−1.32** (−3.00)	2.92	1.03	2.73		GI, GAI
*qA*2	1	107.0	4.69	1.03	SNP157 (104.4)	SNP110* (107.8)	−0.06	**0.66** (3.00)	0.50	0.14	1.27	0.39	Cluster 1	FKF1
*qA*3	2	0.0	3.05	2.99	msat2.5* (0.0)	msat2.5* (0.0)	−0.01	−0.19	**−1.44** (−3.00)	0.00	0.11	3.22		RGA
*qA*4	3	0.0	13.39	3.60	SNP105* (0.0)	nga172* (2.9)	**1.16** (1.80)	0.53 (2.00)	**1.37** (3.00)	52.03	0.80	2.94		RGL2, GAr2
*qA*5	4	3.0	43.23	53.84	msat4.41 (0.0)	FRI* (3.6)	**0.41**	**−2.80** (−6.00)	**−5.62** (−11.40)	6.50	22.84	49.46	Cluster 2	FRI, LD, GA1
*qA*6	4	49.0	8.51	7.38	SNP295* (46.6)	SNP199 (49.9)	−0.03	**−1.95** (−2.60)	**−1.01**	0.03	11.08	1.60	Cluster 3	PHYD
*qA*7	5	19.0	13.67	5.97	SNP136* (17.3)	SNP358* (20.6)	**−0.75** (−1.6)	**−1.39** (−3.40)	**−1.74** (−2.8)	21.75	5.63	4.74	Cluster 4	TFL1,ATMYB33, FLC, CO, TFL2
*qA*8	5	33.0	5.87	6.38	SNP236* (32.1)	nga139 (33.5)	0.03	−0.50	**−2.09** (−5.00)	0.02	0.71	6.84		
*qA*9	5	93.0	8.00	5.43	SNP101 (92.5)	SNP304 (93.8)	**−0.56**	**1.38**	−0.51	12.13	5.55	0.40	Cluster 5	LFY,ELF5, VIN3

a Phenotypic variance explained by each QTL;

b Markers with asterisks were significant in individual family mapping, reported by El-Lithy et al. (2006);

c Values in brackets are two allele's effects (i.e., 2*a*) estimated from individual family mapping.

d Annotations according to reports by Brachi et al. [26], and Ehrenreichet al. [25]. In individual family mapping of L*er*×An-1, *qA*7 was mapped at SNP 130 [21], about 6.1 cM from SNP358 (Fig. S3). The significant effects from JICIM are in bold.

Most loci with significant peaks in the LOD profile have been detected by QTL mapping in individual families [21]. But the LOD scores (Fig. 1) are much higher than those from individual family mapping [21], which empirically demonstrated the higher mapping power of joint linkage analysis. Four of the nine QTL identified in the *Arabidopsis* population, i.e., *qA*1 (GI/GAI) -*qA*3 (RGA) and *qA*8 in Table 1, had significant effects in only one family. Therefore, *qA*1-*qA*3 and *qA*8 were rare QTL in the *Arabidopsis* population. Three of the nine QTL were significant in two families, i.e., *qA*4 (RGL2) detected in families L*er*×An-1 and L*er*×Kond, *qA*6 (PHYD) in families L*er*×Kas-2 and L*er*×Kond, and *qA*9 (LFY/ELF5) in families L*er*×An-1 and L*er*×Kas-2. The other two QTL, i.e., *qA*5 (FRI) and *qA*7 (FLC/CO), had significant genetic effects in all three families.

### Simulation results based on identified QTL

**Figure 2:**
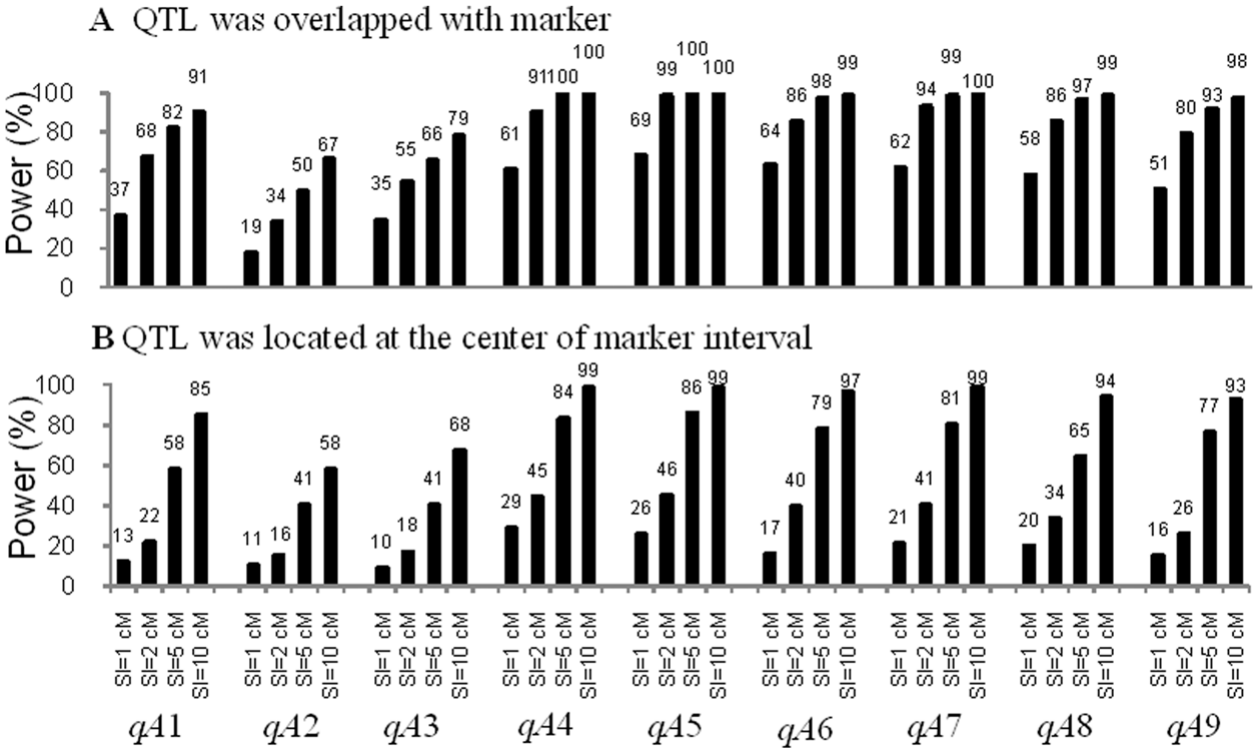
Power of the 9 identified QTL in the *Arabidopsis* population. Power was calculated as the proportion of runs where each QTL was detected within four levels of the support interval (SI = 1 cM, 2 cM, 5 cM, and 10 cM). Two QTL distribution scenarios were considered, i.e., QTL overlapped with markers (A), and QTL at the center of marker intervals (B).

When a QTL overlapped with a marker, the detection power of JICIM was consistently higher in comparison with QTL located in the middle of a marker interval (Table 2 and Fig. 2), since a QTL located midway between markers is the most difficult scenario for QTL detection [27]. As expected, a wider support interval (SI) resulted in higher power.

**Table 2:** Power (%) of the top 30 days-to-silking QTL identified in the maize NAM population under four levels of the support interval, i.e., 1, 2, 5, and 10 cM.

QTL	Chr.	Pos. (cM)	PVE^a^ (%)	QTL overlapped with marker				QTL located at the center of marker interval			
				1 cM	2 cM	5 cM	10 cM	1 cM	2 cM	5 cM	10 cM
*qZ1*	10	41.9	9.55	96.0	97.7	98.4	99.1	76.7	89.6	98.0	98.9
*qZ2*	8	69.7	6.62	95.8	97.4	98.3	98.8	78.8	91.3	97.8	98.7
*qZ3*	9	62.9	6.34	95.2	97.5	98.0	98.8	76.9	91.4	98.1	98.7
*qZ4*	3	56.0	6.25	94.8	97.1	98.0	98.6	77.5	90.5	97.6	98.8
*qZ5*	1	84.6	5.61	96.1	97.4	97.9	98.6	76.3	90.9	97.6	98.8
*qZ6*	2	125.9	5.20	94.2	97.2	98.1	98.9	75.8	90.0	98.5	98.8
*qZ7*	9	57.3	4.33	93.6	97.3	97.9	98.7	74.4	90.0	97.4	98.6
*qZ8*	2	76.5	4.11	93.9	96.7	98.1	98.8	74.2	88.3	97.6	98.8
*qZ9*	3	105	3.88	93.6	97.0	98.1	98.9	74.3	89.0	97.4	98.8
*qZ10*	3	70.6	3.38	93.8	97.9	98.6	99.1	75.7	90.1	97.6	98.6
*qZ11*	7	76.5	3.28	89.8	96.1	98.1	98.7	72.0	89.4	96.7	98.7
*qZ12*	1	137.6	2.79	91.0	96.7	98.2	98.7	73.7	89.2	97.4	98.9
*qZ13*	4	77.2	2.76	89.1	96.4	98.5	99.0	71.7	88.6	97.5	98.8
*qZ14*	2	107.8	2.74	89.4	95.6	98.2	99.2	71.2	87.7	97.2	98.9
*qZ15*	1	181.9	2.73	90.2	96.4	98.2	98.9	71.6	89.2	97.3	98.9
*qZ16*	2	38.6	2.54	91.9	97.4	98.6	99.1	73.4	88.7	97.2	98.7
*qZ17*	5	78.4	2.52	88.8	96.2	98.6	99.2	70.3	87.8	97.8	98.8
*qZ18*	6	86.2	2.51	90.4	96.2	98.1	98.8	71.1	87.3	97.3	98.6
*qZ19*	7	49.2	2.50	91.1	96.7	98.3	99.0	69.8	87.3	97.0	99.1
*qZ20*	3	128.4	2.41	92.3	96.6	98.0	98.5	70.6	87.4	97.1	98.7
*qZ21*	5	101.9	2.35	88.1	95.2	98.4	98.6	71.0	87.3	97.2	98.7
*qZ22*	1	31.7	2.28	89.1	95.8	98.5	99.4	71.2	86.6	97.0	98.7
*qZ23*	4	47.7	2.23	88.4	96.1	98.5	98.9	70.1	87.3	97.7	98.7
*qZ24*	6	27.8	2.06	87.7	95.7	98.4	99.0	68.6	85.6	97.3	98.9
*qZ25*	1	60.8	2.02	88.0	95.1	98.9	99.0	70.4	86.4	97.0	98.6
*qZ26*	8	119.0	2.00	86.5	94.2	97.9	98.8	69.4	87.3	95.7	98.7
*qZ27*	10	82.2	1.95	89.1	96.1	97.9	98.6	69.0	87.4	97.1	98.9
*qZ28*	5	0.0	1.80	88.0	95.0	98.0	98.6	68.3	86.7	96.8	98.6
*qZ29*	8	18.3	1.60	89.1	95.8	98.5	99.3	69.4	86.4	96.3	98.4
*qZ30*	4	111.5	1.37	88.0	95.4	98.6	98.9	70.4	86.4	97.0	98.6

a Phenotypic variance explained by each QTL; QTL were ordered by PVE.

For the maize QTL, detection power was over 95% for all 30 QTL when the length of the SI was 5 cM (Table 2). When we narrowed the SI down to 2 cM, the average power was 96.2% when every QTL overlapped with one marker locus, and 88.8% when every QTL was located in the middle of one marker interval. Even when the SI was 1 cM, we still achieved an average power of 90.9% when the QTL overlapped with markers, and an average power of 72.7% when the QTL was located in the middle of a marker interval. In other words, JICIM places the detected QTL within 1 cM of the true position of the QTL at about a frequency of 90% of the time in the maize NAM population, if the actual QTL is tagged and genotyped in the study. However, the expectation of genotyping of the actual functional polymorphism in this framework is not very realistic, considering the low density of markers used for QTL mapping. Similarly, Guo et al. [28] found functional markers segregating in at least five families and explaining >5% of the phenotypic variance can be detected with adequate power.

In the *Arabidopsis* population, detection power was lower (Fig. 2) than that in the maize population (Table 2), especially when SI = 1 cM, 2 cM, and 5 cM, indicating the strength of using more founders and a larger population size in the maize NAM population. The power of *qA*1-*qA*3 was much lower than that of *qA*4-*qA*9 regardless of the simulated QTL position and the length of the SI. For *qA*4-*qA*9 and when SI> = 5 cM, power was over 90.0% when the QTL overlapped with markers (Fig. 2A). When the QTL was located in the middle of a marker interval, power was over 80.0%, except for *qA*8 (Fig. 2B), which had significant effect only in the third family of the *Arabidopsis* population. These results indicated that for joint linkage mapping, rare QTL are more difficult to detect than common QTL. In the worst case, when *qA*2 was located in the middle of the marker interval and SI = 1 cM, detection power was only 10.7% (Fig. 2B), due to the small additive effect detected in just one family, and only 1.9% of the phenotypic variance was explained (Table 1).

### Detection power of rare QTL in NAM populations

**Figure 3:**
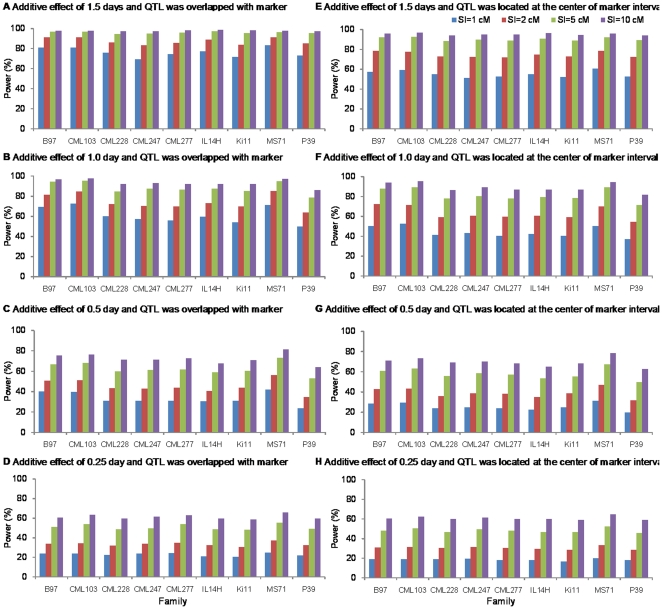
Power of the 9 simulated rare QTL in the maize NAM population. Two scenarios of QTL positions were simulated, i.e., QTL overlapped with markers (A–D), and QTL located at the center of marker intervals (E–H). Four levels of additive effects (1.5 days for A and E, 1.0 day for B and F, 0.5 day for C and G, and 0.25 day for D and H), corresponding to PVE = 13.76%, 6.12%, 1.53%, and 0.38%, were considered. Power was calculated as the proportion of runs where QTL were detected within four levels of the support interval (SI = 1 cM, 2 cM, 5 cM, and 10 cM).

When rare QTL overlapped with markers and their additive effects were greater than half a day (Figs. 3A and 3B), we had a power of more than 50.0% to map QTL within 1 cM SI, 60.0% within 2 cM SI, 75.0% within 5 cM SI, and 85.0% within 10 cM SI. Much lower power can be seen in Figs. 3C and 3D for smaller-effect QTL. For example, when a rare QTL had an additive effect of 0.25 day (PVE = 0.10%) in the B73×Il14H family in the maize population and SI = 1 cM, power was 21.2% when QTL overlapped with markers. When QTL were located in the middle of marker intervals, power was lower than when QTL overlapped with markers (Fig. 3). The reduction in power depends not only on the genetic effect of rare QTL, but also on the length of the SI.

Regardless of whether the QTL overlapped with markers or were located in the middle of marker intervals, the variance of power across families was low (3.9×10^−5^∼0.018; Fig. 3), indicating that similar detection power can be achieved regardless of the family in which the rare QTL was segregating. For QTL having a half-day or a one-day genetic effect, power in family B73×MS71 was slightly higher than that in other families. This was due to the relatively small phenotypic variance observed in family B73×MS71 [5], which led to decreased background noise and increased power.

We also compared the power of rare QTL from JICIM and from individual family mapping. In individual family mapping, we used the family in which a rare QTL was segregating. As an example, a rare QTL was assumed to be segregating in family B73×Il14H with various genetic effects (i.e., 0.25, 0.5, 1.0, and 1.5 days; Table 3) and PVE = 0.10%, 0.39%, 1.55%, and 3.49% for the whole maize NAM population, respectively. For a QTL with more than a 1-day effect, power from both joint linkage mapping and individual family mapping was consistently high, while individual family mapping had higher power when the length of the SI was less than 5 cM. Moreover, individual family mapping was able to narrow the SI down to 1 cM with a power of 74.5%, but 59.9% for JICIM when the QTL overlapped with markers. However, for a QTL with less than a 1-day effect, the reverse happens. Power from joint linkage mapping, despite being low, was slightly higher than that from individual family mapping. Power decreased sharply for QTL with a 0.25-day effect from individual family mapping, to below 15.0% in all cases. Similar results were observed for rare QTL segregating in other families.

**Table 3:** Simulated power (%) from JICIM and ICIM for rare QTL segregating in family B73×Il14H under four levels of support interval, i.e., 1, 2, 5, and 10 cM.

Method	Additive effect (day)	QTL overlapped with marker				QTL located at the center of marker interval			
		1 cM	2 cM	5 cM	10 cM	1 cM	2 cM	5 cM	10 cM
JICIM in the maize NAM population	1.50	77.4	89.1	97.5	98.6	55.2	75.0	91.0	96.4
	1.00	59.9	73.2	87.6	92.0	42.6	60.5	79.7	87.1
	0.50	30.4	40.8	58.8	67.7	22.6	35.1	53.3	64.9
	0.25	21.2	32.5	48.6	59.8	18.1	29.7	46.9	59.9
ICIM in family B73×Il14H	1.50	84.7	92.2	94.8	94.8	51.6	71.2	87.1	93.5
	1.00	74.5	84.7	93.2	94.4	44.4	61.7	80.6	87.1
	0.50	25.3	42.4	53.2	61.0	21.9	33.3	49.5	57.3
	0.25	4.9	6.8	8.8	11.9	3.0	4.0	7.1	10.5

### Dissection of linked QTL

To investigate potential confounding when QTL are in proximity of each other, we used *qZ*6 and *qZ*8 identified in the maize NAM population to design the simulation experiment. In the actual population, they were located on chromosome 2, 49.4 cM apart, and explained 5.20% and 4.11% of the total phenotypic variance, respectively (Table 2). The genetic model used in simulation contained only the effects of *qZ*6 and *qZ*8, and no background genetic effects were included. Correlation of their effects across populations is 0.21.

**Figure 4:**
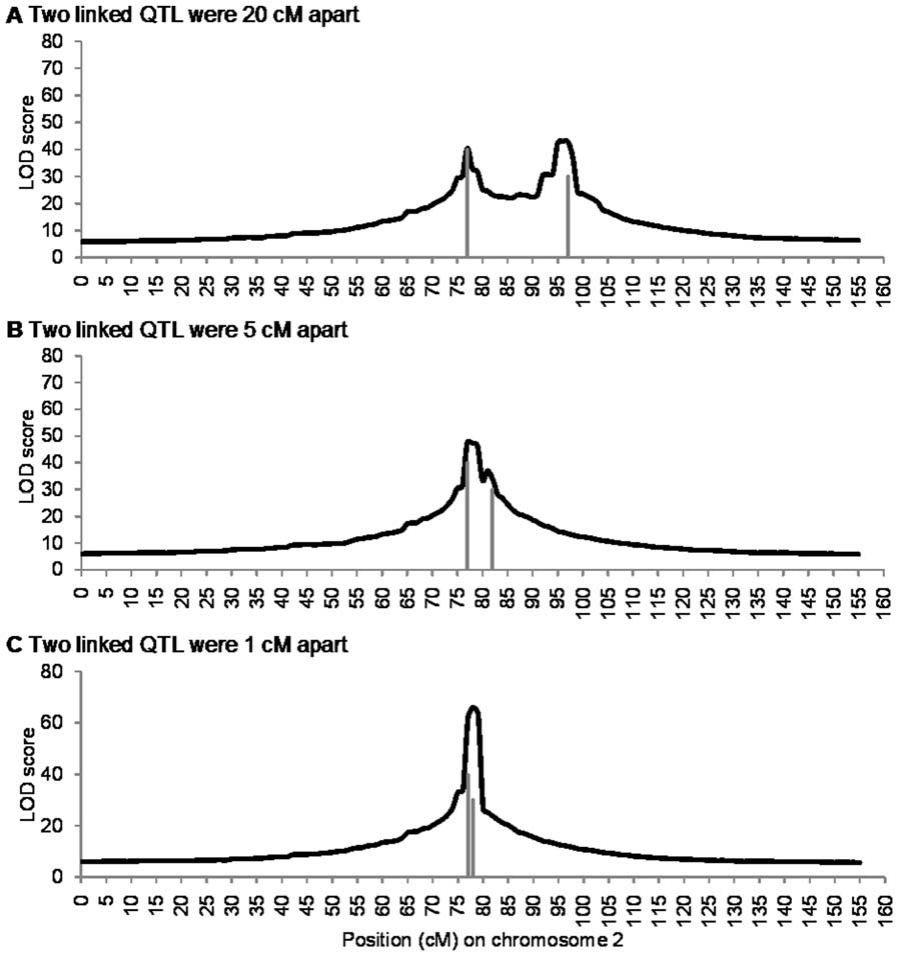
Mean LOD profile of the second chromosome from 100 simulation runs. Two linked QTL were respectively located at 77 cM and 97 cM (A), 77 cM and 82 cM (B), and 77 cM and 78 cM (C) on chromosome 2. The two vertical lines indicate the two QTL. The length of each line was proportional to the size of its corresponding QTL. The mean LOD profile of the other chromosomes was almost equal to zero, and thus was not shown.

**Figure 5:**
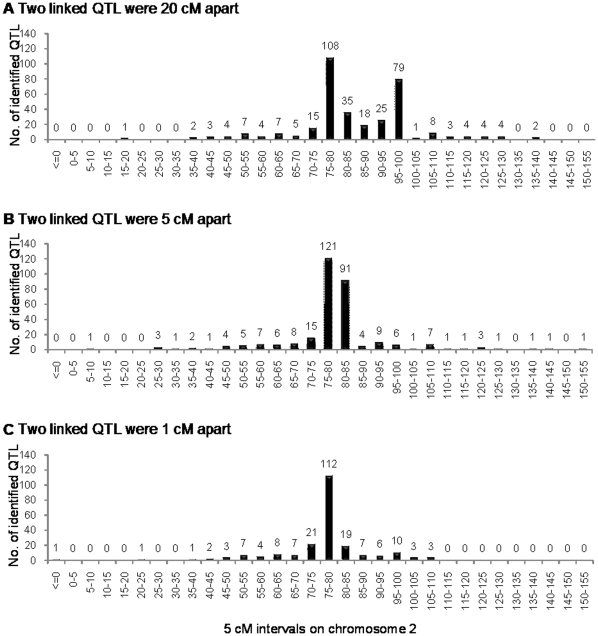
Number of identified QTL in each marker interval on the second chromosome. Two linked QTL were respectively located at 77 cM and 97 cM (A), 77 cM and 82 cM (B), and 77 cM and 78 cM (C) on chromosome 2. The LOD threshold was 12.26.

When *qZ*6 and *qZ*8 were putatively located at 77 cM and 97 cM, from the mean LOD profile we can see that there were two clear peaks around the putative positions of *qZ*6 and *qZ*8 (Fig. 4A). The number of identified QTL was 108 within 75–80 cM and 79 within 95–100 cM (Fig. 5A). The additive effects for *qZ*6 and *qZ*8 were slightly overestimated (Table S2). All simulated populations showed that *qZ*6 and *qZ*8 can be mapped as two distinct QTL. When they were closer, say 5 cM apart, the two LOD peaks were closer as well (Fig. 4B). The numbers of identified QTL around *qZ*6 and *qZ*8 were still very high, i.e., 121 and 91, respectively (Fig. 5B). From the individual LOD profiles (Table S3), nearly 70% of the simulated populations can separate *qZ*6 and *qZ*8 properly. When *qZ*6 and *qZ*8 were 1cM apart, only one peak appeared in the mean LOD profile (Fig. 4C), and 112 QTL were detected within 75–80 cM (Fig. 5C). The estimated effects at the peak were almost equal to the sum of the two QTL effects (Table S2), indicating the two linked QTL were detected as one single QTL at an intermediary position.

## Discussion

### Efficiency of JICIM for complex trait dissection in the NAM design

In the maize and *Arabidopsis* NAM populations, the LOD score of JICIM was much higher than that from individual family mapping, indicating the high mapping power of JICIM. In the maize population, JICIM had a more than 85% chance of locating QTL within 1 cM SI when QTL overlapped with markers, and a more than 68% chance when QTL were located in the middle of marker intervals (Table 2). For linkage mapping in a biparental population with a size of hundreds, the mapping resolution is always in the 10 cM magnitude due to limited number of recombination that can be surveyed in such population sizes, not to mention that the effect sizes are usually grossly overestimated in such studies. Cross-validation was suggested as a viable option for diminishing the effects of such sampling and resolution issues. In such populations, only if an individual QTL is explaining more than 10% of phenotypic variance, a resolution below 5 cM can be achieved [2], and rigorous testing via bootstrapping and other re-sampling strategies are necessary for accurate estimation of the effect sizes. In addition, although not comparable to the scale of association mapping in diverse populations where hundreds of alleles per locus can be simultaneously tested, in JICIM multiple alleles at the order of tens can easily be tested simultaneously (Tables S1 and 1).

Flowering time is an adaptive trait that is highly correlated with population structure, as demonstrated in maize [29] and *Arabidopsis* [30]. Joint linkage mapping of NAM populations better avoids the confounding effect of population structure, and identified most genetic variation of flowering time (Tables S1 and 1). Thus joint linkage mapping provides us with a promising approach to study the genetics of flowering time.

### Efficiency of the NAM design for rare QTL detection

The power of QTL detection in a mating design involving multiple crosses depends not only on QTL effects, but also on allele frequency (Table 2; Fig. 2). For the maize population, high detection power has been observed for QTL segregating in 2–4 families (Tables S1 and 1). The power of rare QTL was around 80% for 3-day effects in the *Arabidopsis* population (Fig. 2), and for a 1-day effect in the maize population (Fig. 3) within 5 cM SI. We were interested in finding out whether it is more efficient to use individual family mapping for rare QTL. Simulation results showed that to map rare QTL with more than a 1-day effect, joint linkage mapping was not of merit, since adding more families with non-significant QTL would increase the sample variance; this, in turn, could cause the QTL signals to become vague, and the position of the QTL could be distorted by neighboring QTL in other families. For small-effect QTL, the power of individual family mapping was slightly lower than the power of joint linkage mapping. One possible reason may be the actual genetic architecture used in simulation as the background effect. In maize NAM, over 98% of the QTL alleles had less than a 1-day effect on days to silking ([5] and Fig. S1). In this sense, the background effects from other families may be helpful for rare QTL with less than a 1-day effect. Considering the experiment expense and the statistical work load of joint linkage mapping, individual family QTL mapping is still useful, especially for rare QTL.

### Integration of linkage maps in NAM populations for joint QTL mapping

When building an integrated linkage map for joint linkage mapping or joint association-linkage mapping, variation in recombination frequency among families is commonly observed in some chromosome regions [6]. To avoid the confounding effect of this variation, JICIM recognizes the recombination frequencies across families by recalculating the crossovers in each family. To accentuate the situation further, Buckler et al. [5] found an association between flowering time QTL with larger effects and reduced local recombination rates in the maize NAM population. This is not completely unexpected since flowering time is a characteristic life history trait that may have influence over the species evolution. For regions around *vegetative to generative transition 1* (*Vgt1*) locus on chromosome 8 [31], recombination frequencies were significantly different across QTL effects estimated in each family and across the population structure of the 26 parental lines [6]. Under such circumstances where there are more sources of confounding for the estimation of the QTL effect sizes, stepwise regression seems to provide a more accurate effect estimation (see Fig. 5 in Buckler et al. [4]) than JICIM (Table S1). However, once the marker density across the *Vgt1* region is increased, JICIM increase the mapping resolution and give a much more accurate position and effect estimation of *Vgt1*. At the same time, another early flowering QTL located about 5 cM upstream from *Vgt1* [32] was confirmed by JICIM in the maize population (Fig. S2).

### Further considerations

Simulations based on putative genetic models are commonly used to investigate the efficiency of a QTL mapping method, where QTL detection power and false positive rate can both be properly estimated [33]–[36]. But there is a concern whether the predefined genetic model could represent the realistic model. Simulations in this study used real phenotypic data as the background effect, which represents the realistic model. In this case, it is less likely to distinguish the false positives from the true positives [37]. Instead, the genome wide 2-error rate was used to control the false positives. The power thus determined could be overestimated when the moving QTL is in the vicinity of the previously identified QTL. The simulation study where the residual of the linear model (details in Material and Methods) is used as the background may provide more convinced power.

In this study, we only considered the QTL fusion of two identified QTL under three linkage distances. To make more inclusive conclusions, further studies will be needed by including more factors, such as linkage distance, correlation of two QTL effects across populations, two QTL segregating in the same populations or different population, and the allelic effects that are correlated positively or negatively across populations, etc.

We used multiple families sharing one common parent to illustrate the algorithm of JICIM. Theoretically, there is no limit to how much JICIM may be extended to other mating designs involving multiple crosses, such as the eight-way cross [38] and the diallel cross [39]. In biparental populations, ICIM is able to detect dominance [36] and digenic epistasis [35]. Further studies are needed to extend JICIM to other mating designs and genetic models including dominance and epistasis. In order to maximize QTL mapping power, comparison of different mating designs, and the balance between the number of families and the number of individuals in each family of a NAM design, can be further investigated by JICIM.

## Materials and Methods

### Statistical models in joint QTL mapping

For multiple crosses sharing one common parent, we assume that there are *F* (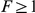) families with a size of 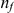 individuals in the *f* ^th^ family, and a total population size of *N* (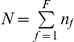). The proposed statistical method consists of two steps. In the first step, GLM was used where population and population-by-marker interactions were treated as fixed effects. Each marker has *F*+1 levels (the common parent and the other *F* founders) in the genetic linkage map. These parameters were included in the following model,
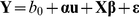
(1)


where **Y** is the vector of phenotypic values; 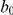 is the intercept; 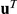 *= *(*u*_1_, *u*_2_, *…, u_F_*) is the effect vector indicating the cross effect of each founder with the common parent; 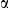 is the *N*×*F* incidence matrix relating each *u_f_* (*f* = 1, 2, …, *F*) to **Y**; 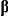 is the [(*F*+1)*m*]×1 effect vector of the *N*×[(*F*+1)*m*] incidence matrix **X** where *m* is the number of markers; and 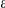 is the vector of residuals. To avoid over-fitting, we used stepwise regression to estimate the parameters in model [1]. If the regression variable did not enter into the model, the corresponding coefficient was set at 0.

Based on coefficient estimation, one-dimensional scanning is conducted in the second step, which is similar to the second step of inclusive composite interval mapping (ICIM) [33], [34]. At a testing position in a marker interval (*k*, *k*+1), all individuals in the *f* ^th^ family can be classified into four groups based on the allelic states of the two flanking markers (Table 4). All missing marker types were assigned to either the common parent genotype, or the non common parent genotype through an imputation algorithm that considers linkage relationship between markers within each family [40]. Phenotypic value in model [1] can be adjusted by the estimated coefficients in the first step to exclude the influence of QTL on other chromosomal regions, i.e.,
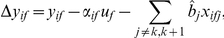



**Table 4:** Genotypic distribution of QTL in the *f* ^th^ family under four marker types in mapping interval (*k*, *k*+1), adapted with modification from Table 1, Li et al. (2007).

Group	Sample size	Frequency	Marker genotype		Frequency of QTL genotype		Distribution of 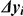
			*k*	*k*+1	*Q_f_Q_f_*	*q*_0_ *q*_0_	
1	*n_f_*_1_	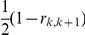	+	+	*p_f_*_1_	1- *p_f_*_1_	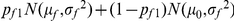
2	*n_f_*_2_	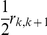	+	-	*p_f_*_2_	1- *p_f_*_2_	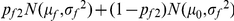
3	*n_f_*_3_	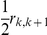	-	+	*p_f_*_3_	1-*p_f_*_3_	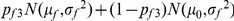
4	*n_f_*_4_	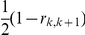	-	-	*p_f_*_4_	1-*p_f_*_4_	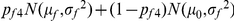

Note: 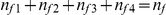, where *n_f_* is the size of the *f* ^th^ family. *Q_f_* is the allele in the *f* ^th^ parent, and *Q*_0_ is the allele in the common parent. 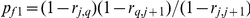, 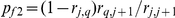, 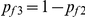, and 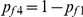, where 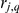, 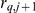 and 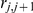 are the recombination frequencies in the *f* ^th^ family between marker *j* and the putative QTL, between the putative QTL and marker *j*+1, and between markers *j* and *j*+1, respectively. “+” and “-” denote two types of homozygote for the marker genotype. 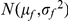 and 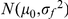 represent the distributions for the two QTL genotypes *Q_f_Q_f_* and *q_0_q_0_* in the *f* ^th^ family, respectively. All missing marker types were assigned to either the common parent genotype, or the non common parent genotype through an imputation algorithm that considers the linkage relationship between markers within each family [37]; thus this table assumes no missing genotypic data.

where *i* = 1, 2, …, *n_f_*, *f* = 1, 2, …, *F* and the current scanning position is within the interval determined by the *k* ^th^ and *k*+1^th^ marker.

If there is a QTL (with the two alleles in the *f* ^th^ family denoted as *Q_f_* and *Q*_0_) at the testing position, 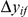 (*i* = 1, 2, …, *n_f_*) follows a mixture distribution consisting of the distribution of 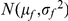 for individuals with QTL genotype *Q_f_Q_f_*, and the distribution of 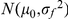 for individuals with QTL genotype *Q*_0_ *Q*_0_. Proportions of the two component distributions depend on the recombination frequencies between the QTL and its two flanking markers. The existence of a QTL at the current mapping position can be tested by the following hypotheses: 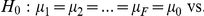




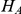
: at least one of 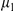, 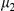, …, and 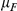 not equal to 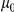.

The log-likelihood function under the alternative hypothesis *H_A_* is,
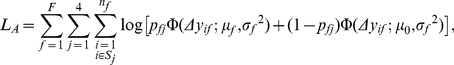
(2)


where *S_j_* denotes the *j* ^th^ marker type group (*j* = 1, 2, 3, and 4; Table 4); *p_fj_*, the proportion of QTL genotypes *Q_f_Q_f_* in the *j* ^th^ marker group of the *f* ^th^ family, was calculated from the recombination frequencies between two flanking markers in the current scanning interval, and between either side of the flanking marker and the putative QTL located between the interval; 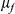 and 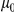 are the means for QTL genotypes *Q_f_Q_f_* and *Q*_0_ *Q*_0_, respectively, and 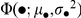 represents the probability density of the normal distribution. The expectation and maximization (EM) algorithm was used to estimate the *F*+1 means and *F* variances in equation [2], and their maximum likelihood estimates are represented by 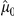, 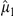, 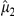,…, 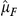, and 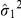, 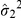,…, and 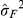, from which the additive effect of the putative QTL in each family (denoted by 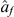) can be estimated as 
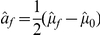
.

In QTL mapping for a bi-parental population, when we calculated *p_fj_*, the recombination frequencies between the two flanking markers in the current scanning interval and between either side of the flanking marker and the putative QTL located within the interval were estimated from the marker positions in the linkage map through mapping function, such as the Morgan, Haldane, and Kosombi function, etc. [41]. To deal with the variation in recombination frequency across families in joint linkage mapping by a consensus linkage map, recombination frequencies were estimated from the observed crossovers in the *f* ^th^ family.

Under the null hypothesis *H*_0_, 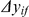 (*i* = 1, …, *n_f_*) within each family follow a normal distribution denoted as 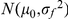. The mean and variance of this distribution can be estimated as,
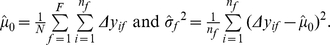



Thus, the maxima log-likelihood function under the null hypothesis *H*_0_ is,
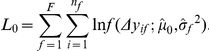



The LOD score at the testing position can be calculated from the log-likelihoods under the two hypotheses. If LOD score decays at least one in both sides of the higher-than-LOD threshold peak, the position corresponding to that higher-than-LOD threshold peak is declared as the existence of a QTL. In this sense, each QTL was identified by its one-LOD support interval.

### Phenotypic variance explained by each QTL

In a biparental population, such as a RIL population, the theoretical genetic variance for each QTL is *a* ^2^, where *a* is the additive effect of that QTL, defined as half of the difference between the two homozygous QTL types. Ignoring segregation distortion, the phenotypic variance explained (PVE) is,
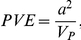



where *V_P_* is the phenotypic variance observed in the biparental population. For the NAM design, assuming there are *F* families, the genotypic value of the common parent QTL genotype and those of the other *F* parents are 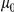, 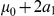, …, 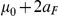, respectively, and the corresponding frequencies of these *F*+1 genotypic values are 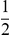, 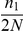, …, 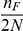, respectively. Therefore, the genetic variance of the *F*+1 QTL genotypes in the NAM population can be calculated as,
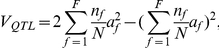



Thus, 
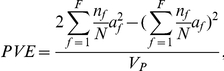



where 
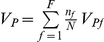
 and *V_Pf_* is the phenotypic variance of the *f* ^th^ family. The phenotypic variance used here is the weighted average of variances of the *F* families. The difference between family means contributes to the phenotypic variance of the whole NAM population, which is not suitable to calculate PVE of any QTL in the NAM population. It can be proved that the weighted variance of the *F* families is equal to the variance of the NAM population adjusted by family means. It should be noted that the total genotypic variance of all the identified QTL cannot be viewed as the sum of genotypic variance of individual QTL, due to the possible linkage between them.

### The maize and *Arabidopsis* NAM populations

The maize NAM population has 25 biparental families sharing the common parent B73. From each cross, 200 RILs were produced, resulting in a total of 5000 lines. 4699 RILs and 1106 markers were used to construct the consensus linkage map, which has a total length of 1400 cM, and one marker every 1.3 cM on average [6]. All the genetic markers show polymorphism between B73 and all the other 25 parents. The best linear unbiased prediction of days to silking for each RIL across environments [5] was used as the phenotypic data in joint QTL linkage mapping.

To give another example, we combined three *Arabidopsis* RIL families [21], i.e., L*er* (Landsberg *erecta*) ×Antwerp (An-1), L*er* × Kashmir (Kas-2), and L*er* × Kondara (Kond), in a multiple cross design sharing one common parent. For our purposes, we only obtained the raw marker and phenotype data from the original study, and reconstructed the genetic maps and QTL scans. A total of 109 genetic markers showed polymorphism between L*er* and the other 3 parents. A similar strategy as used in the maize population [6] was adopted to construct the consensus map in the *Arabidopsis* population by MAPMAKER 3.0 [42]. The L*er* allele was designated as the “A” parent allele, the other three parent alleles were designated as the “B” parent alleles, and heterozygous loci were converted to missing data. In addition, markers that were non-polymorphic in a particular family were converted to missing data. (Marker SNP395 has a chromosomal conflict, since it could be located on either chromosome 4 or 5; therefore this marker was not used for further joint linkage mapping.) The consensus map thus obtained consists of 108 marker loci, and has a total genetic length of 499.7 cM and an average marker density of 4.81 cM (Fig. S3).

### Model selection criteria in JICIM

Permutation tests were conducted using days to flowering to determine the criteria of model selection in the first step of JICIM. To permute the data, overall and population means were fitted to the original data, and predicted values and residuals were calculated. The residual was randomized, and then added back to the predicted value [43]. Associations of all markers for the updated dataset were re-calculated and the lowest *P*-value for all markers was identified. This procedure was repeated 1000 times to establish a distribution of *P*-values to test the null hypothesis. For both populations, the *P*-value corresponding to the overall type I error 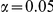 was approximately 10^-4^. Thus in the first step of JICIM, we used *P* = 10^−4^ as the probability for markers entering into the model, and *P* = 2×10^−4^ as the probability for markers moving out of the model.

### Power simulation of the identified QTL

To determine the reliability of identified QTL and the mapping resolution of JICIM, genetic effects and allele frequency in simulation were the same as those of QTL identified in the maize or *Arabidopsis* population, which represented the general properties of the observed genetic architecture. To avoid simplifying simulated model, and to simulate the genetic scheme as genuinely as possible, we used the real phenotype as the background, that is,
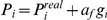



where *i* = 1, 2, …, *n_f_*, *P_i_* is the simulated phenotype for the *i* ^th^ individual in the *f* ^th^ family, *P_i_^real^* is the real phenotype for the *i* ^th^ individual in the *f* ^th^ family, *a_f_* is the additive effect of one of the identified QTL in maize or *Arabidopsis* in the *f* ^th^ family, and *g_i_* is the genotype of the identified QTL for the *i* ^th^ individual in the *f* ^th^ family.

We considered two scenarios of QTL distribution in our simulation experiments. In the first scenario, the number of simulation runs was equal to the number of markers, i.e., 1106 for the maize population, and 108 for the *Arabidopsis* population. In the first simulation run, the QTL was located at the first marker locus; in the second run, the QTL was at the second marker locus; and so on. Thus, for each individual, the QTL genotype was the same as its overlapped marker. In the second scenario, the number of simulations was equal to the number of marker intervals, and QTL were assigned to the middle of marker intervals. In the first simulation run, the QTL was located in the middle of the first marker interval; in the second run, the QTL was in the middle of the second marker interval; and so on. Thus the QTL genotype has equal probabilities of being the genotype on either side of the two flanking markers. That is, the QTL genotype was assigned as the genotype of left flanking markers with a probability of 0.5, and as the genotype of right flanking markers with a probability of 0.5. The number of runs was 1096 for the maize population, and 103 for the *Arabidopsis* population.

Each of the top 30 and 9 identified QTL in the maize and *Arabidopsis* populations, respectively, was simulated to determine whether its effect could be detected by the LOD threshold from permutation tests within a pre-fixed length of the support interval (SI) centered at the true QTL position. The proportion of detected QTL across markers or marker intervals was used as the detection power of each simulated QTL. Because simulation in this study used real phenotypic data as the background effect, it was less likely to distinguish the false positives from the true positives in the simulated population [37]. Therefore, we used the genome-wide *k*-error rate (GWER(*k*)) [20] to guard against more than *k* false positives.

### Rare QTL Simulation

To investigate the possibility of incorrectly mapping linked QTL that segregate in only a few populations as a single QTL, we simulated nine rare QTL in maize population, that segregate in one family with four effects, i.e., 1.5, 1.0, 0.5 and 0.25 days, corresponding to PVE = 3.49%, 1.55%, 0.39%, and 0.10%, respectively. The nine rare QTL were assumed to be segregating in families B73×B97, B73×CML103, B73×CML228, B73×CML247, B73×CML277, B73×IL14H, B73×Ki11, B73×MS71, and B73×P39, respectively. Phenotypic variances in these families are 2.61, 2.08, 7.85, 8.26, 9.82, 5.41, 9.17, 2.62 and 5.59, respectively. For each of the 36 combinations of nine rare QTL and four genetic effects, two QTL distribution scenarios were considered for power simulation, as described in the previous section.

### Linked QTL Simulation

To evaluate potential confounding when QTL are located in proximity of each other, we designed additional simulation experiments based on the maize NAM linkage map and two linked QTL explaining similar phenotypic variance in the maize NAM population. To keep constant the phenotypic variance explained by the two QTL, the phenotypic variance of each simulated family was equal to that of the maize NAM population. The error variance in each simulated family was calculated by 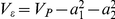, where *V_P_* is the phenotypic variance of the family in the real maize NAM population, and *a*_1_and *a*_2_ are the additive effects of the two linked QTL, respectively. Thus, *a*_1_, *a*_2_, and 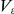 were used to simulate the phenotypic value of the *f* ^th^ simulated family. No background genetic effect was considered, and this was generally adopted to compare different QTL mapping methods [33]–[36]. The principle described above has been used to investigate the effect of population size and marker density on the detection of coupling and repulsive linkage in ICIM [2].

## Supporting Information

Figure S1 **A The distribution of QTL across 25 maize NAM families; B Histogram of additive allele estimates for the 52 days to silking QTLs for 25 founder lines relative to B73.** Count of effects increasing flowering time above the line, and decreasing flowering time below the line.
(TIF)

Figure S2 **LOD score profile for chromosome 8 from the joint inclusive composite interval mapping (JICIM) and general linear model (GLM).** Both JICIM and GLM were implemented when including a miniature transposon (*MITE*) as a marker, which associated a previously identified vgt1 allele from northern germplasm. In this case, *vgt1* were accurately identified, while another gene (*vgt2*) associated flowering time were also identified.
(TIF)

Figure S3 **The consensus linkage map from three RIL families in the** ***Arabidopsis*** **NAM population.**
(TIF)

Table S1 **Fifty-two identified QTL for maize NAM population by JICIM.**
(XLS)

Table S2 **The estimated QTL effects from 100 simulation runs to test QTL confounding.**
(XLS)

Table S3 **LOD profiles on chromosome 2 for the 100 simulation runs when QZ6 and QZ8 were located at 77cM and 82cM on chromosome 2.**
(XLS)
