# Supplementary figures and images for: Joint QTL Linkage Mapping for Multiple-Cross Mating Design Sharing One Common Parent
Source: PLoS One. 2011 Mar 15;6(3):e17573. doi: 10.1371/journal.pone.0017573 (PMC3057965; doi:10.1371/journal.pone.0017573)

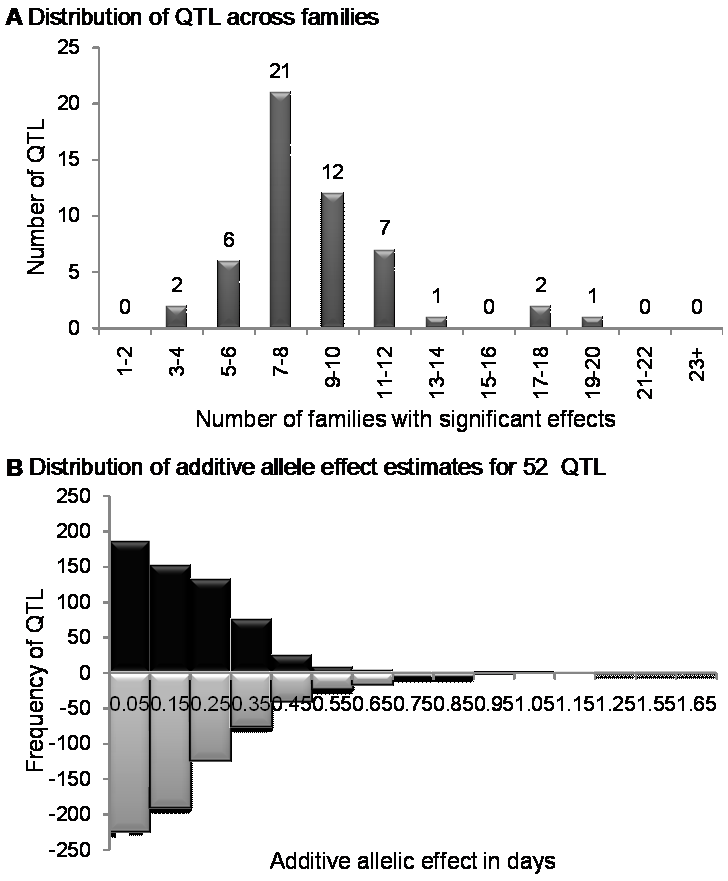

Supplement: Figure S1 — A The distribution of QTL across 25 maize NAM families; B Histogram of additive allele estimates for the 52 days to silking QTLs for 25 founder lines relative to B73. Count of effects increasing flowering time above the line, and decreasing flowering time below the line. (TIF) [file pone.0017573.s001.tif]

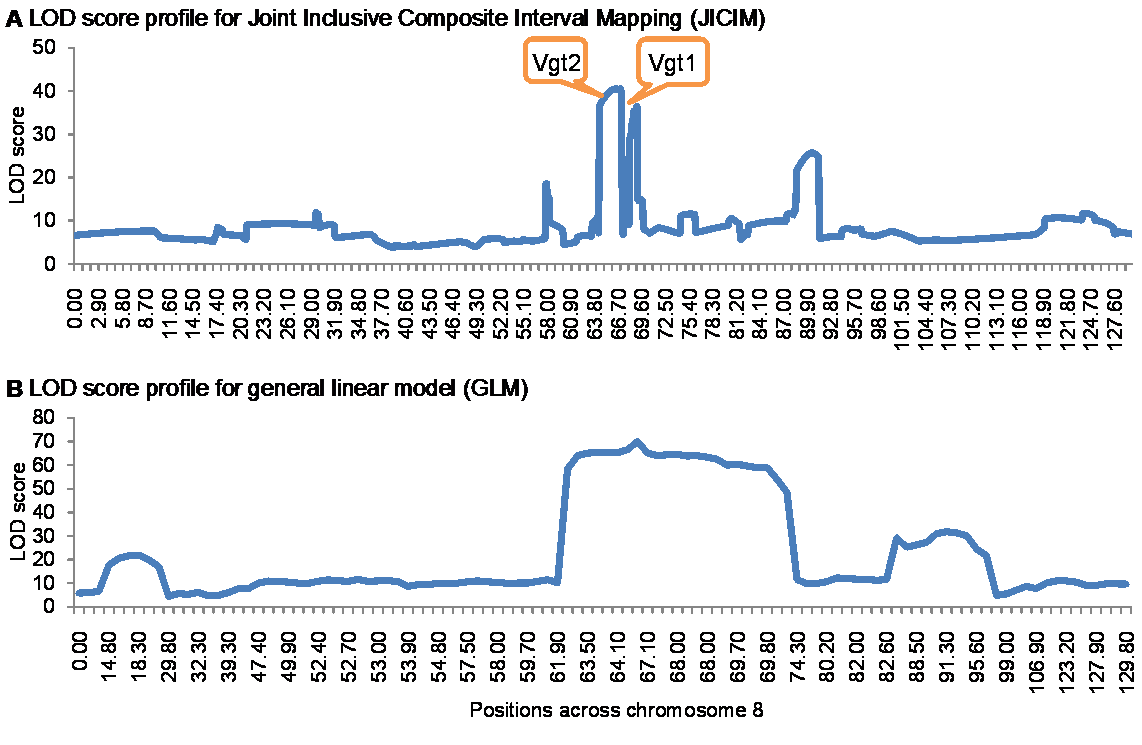

Supplement: Figure S2 — LOD score profile for chromosome 8 from the joint inclusive composite interval mapping (JICIM) and general linear model (GLM). Both JICIM and GLM were implemented when including a miniature transposon (MITE) as a marker, which associated a previously identified vgt1 allele from northern germplasm. In this case, vgt1 were accurately identified, while another gene (vgt2) associated flowering time were also identified. (TIF) [file pone.0017573.s002.tif]

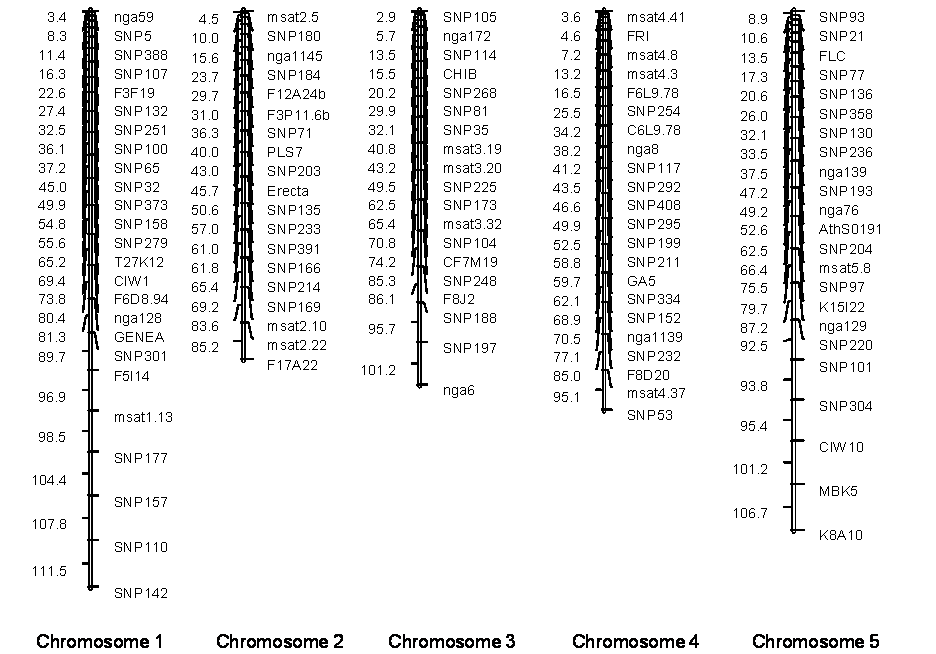

Supplement: Figure S3 — The consensus linkage map from three RIL families in the Arabidopsis NAM population. (TIF) [file pone.0017573.s003.tif]
